# Physiological response to fetal intravenous lipid emulsion in mid-gestation

**DOI:** 10.1042/CS20256946

**Published:** 2025-09-24

**Authors:** Neeka Barooni, Athena Chen, Sarah M. Alaniz, Jessica Minnier, Samantha Louey, Sonnet S. Jonker

**Affiliations:** 1 Center for Developmental Health, Knight Cardiovascular Institute, Oregon Health & Science University, Portland, OR, U.S.A; 2 Department of Pathology, Providence Portland Medical Center, Portland, OR, U.S.A; 3 School of Public Health, Oregon Health & Science University, Portland, OR, U.S.A; 4 Knight Cardiovascular Institute, Oregon Health & Science University, Portland, OR, U.S.A

**Keywords:** fetus, Intralipid, lipid metabolism, parenteral nutrition, preterm, sheep

## Abstract

Circulating lipid levels are typically low in fetuses, and exposure to high lipid levels at developmental stages prior to term birth is sometimes associated with pathology. Experimentally, near-term fetuses tolerate one week of high lipid concentrations; it is unknown whether this brief exposure to elevated circulating lipids is pathological at an earlier developmental age. We studied the physiological response to intravenous lipid emulsion during mid-gestation. Fetal sheep received intravenous Intralipid 20® (*n* = 9) or Lactated Ringer’s Solution (*n* = 8) from 85.0 ± 0.7 to 97.0 ± 0.7 days of gestation (term = 147 days). Intralipid was administered according to manufacturer’s recommendations, with an initial dose of 0.5–1 g/kg/d that increased daily to a maximum of 3 g/kg/d. Hemodynamic and arterial blood parameters were assessed throughout the study. Fetal growth, liver function, and lipid droplet accumulation were measured on the final day. Fetal hemodynamics and blood gases did not change as a result of the treatment. Compared with Controls, Intralipid fetuses had lower blood lactate concentrations (1.3 ± 0.2 vs. 1.0 ± 0.2 mmol/l, *P*=0.009) after eight days of treatment. Conjugated (0.4 ± 0.1 vs. 0.6±0.1 mg/dl, *P*<0.001) and unconjugated (0.3 ± 0.1 vs. 1.2 ± 0.5 mg/dl, *P*<0.001) bilirubin levels were higher in Intralipid-infused fetuses than in Controls. Fetal somatic growth was unchanged, but heart weight was lower in fetuses receiving Intralipid (6.9 ± 0.7 vs. 6.1±0.7 g, *P*=0.008). Compared with Controls, Oil Red O staining was elevated in the liver and heart of Intralipid-infused fetuses (liver score: 18.9 ± 17.2 vs. 371.7±44.2, *P*<0.0001; heart score: 1.8 ± 2.8 vs. 97.6 ± 60.1, *P*=0.0006). Our findings suggest that mid-gestation fetal sheep can tolerate intravenous lipid emulsion. Lipid accumulation in the liver and heart may precede pathologies associated with ectopic lipid storage, but further research is needed to understand the long-term consequences of Intralipid infusion at this developmental stage.

## Introduction

Circulating lipid concentrations are typically low during fetal development. Tissues primarily use more readily available fuel sources such as glucose and lactate *in utero* [1–4]. However, lipid levels can be prematurely elevated by maternal dyslipidemia [5,6], infection during pregnancy [7], or premature birth and the administration of parenteral nutrition. Current guidelines recommend that all neonates less than 37 weeks receive parenteral nutrition for several weeks or months after birth [8,9]. This often includes parenteral lipid emulsions, which raise circulating concentrations of triglycerides, fatty acids, and cholesterol considerably in preterm infants [10–13]. How premature elevations in circulating lipids affect the physiology throughout development is not well studied.

Consequences of premature exposure to high circulating lipids include metabolic dysregulation, impaired organ growth, and vulnerability to cardiovascular dysfunction later in life [10,14–20]. Previous studies suggest that Intralipid treatment contributes to the increased risk for hypertriglyceridemia, jaundice, parenteral nutrition-associated liver disease, pulmonary arterial lipid accumulation, aortic stiffness, impaired cardiac function, and sepsis in premature infants [9,17,18,21,22]. Yet, the mechanisms underlying these conditions are poorly understood as the study of elevated lipids in the fetus is challenging, and it is likewise difficult in preterm infants to understand outcomes resulting from premature lipid exposure versus pathologies related to preterm birth.

In the present study, we infused Intralipid into fetal sheep from 89 to 97 dGA (61–66% of gestation) to isolate the physiological response to elevated lipid concentrations during mid-gestation. Our recent findings indicate that near-term (85–90% of gestation) fetal sheep are relatively tolerant of Intralipid emulsion [23,24]. However, less mature fetuses are at greater risk for hyperlipidemia due to lower rates of lipid clearance, immature fat oxidation systems, and limited adipose tissue availability [25,26]. We collected fetal plasma on the first, fourth, and eighth day of treatment to measure circulating concentrations of cholesterol, triglycerides, and phospholipids. Additionally, we characterized fetal hemodynamics, arterial blood chemistry, and circulating hormone levels before and after the eight-day infusion period. On the final study day, we analyzed fetal growth, liver function, and lipid droplet accumulation in the heart, liver, lungs, and placenta.

## Methods

### Animals

All animal experiments were conducted at Oregon Health & Science University, which is accredited by AAALAC International, and approved by the Institutional Animal Care and Use Committee (#IP0007). Healthy timed-bred ewes in good body condition carrying twin fetuses were obtained from a local supplier. Ewes underwent sterile surgery at 85.0 ± 0.7 (mean ± SD) days of gestational age (dGA) to place fetal arterial and venous catheters as previously described [23,24] and to place single catheters (0.58 mm inner diameter x 0.99 mm outer diameter) in one jugular vein and one carotid artery. Ewes were induced for surgery with ketamine (4–7 mg kg^-1^ intravenous, Boehringer Ingelheim, Ridgefield, CT) and diazepam (0.1–0.2 mg kg^-1^ intravenous, Hospira, Lake Forest IL), and ventilated with 100% oxygen (~2 L min^-1^). Anesthesia was maintained with isoflurane (up to 2.5%, Piramal, Bethlehem, PA). Ewes received subcutaneous buprenorphine HCl (0.3 mg; Covetrus, OH, U.S.A.) either immediately before or after surgery and sustained release buprenorphine (0.05 mg kg^-1^, Wedgewood Pharmacy, NJ, U.S.A.) after surgery. Surgical recovery was four days. Exclusion criteria were fetal congenital abnormalities, hydrops fetalis, death, or deviations from the flock historical 90% confidence interval for fetal body mass, arterial pH, hematocrit, or arterial partial pressure of oxygen (PO_2_). Fourteen ewes were allocated to the study from which 17 fetuses were studied; reasons for exclusion were cord entanglement leading to severe hypoxia and demise, catheter failure, and hydrops fetalis.

### Fetal monitoring and experimental model

Ewes acclimated to metabolic crates with free access to food and water and the ability to stand or lie down at will, allowing continuous fetal hemodynamic monitoring and infusion [27,28]. Catheter patency was maintained by continuous very low-volume heparinized Lactated Ringer’s Solution infusion (Minipuls 3, Gilson, Middleton, WI, U.S.A.). Catheters were connected to a bridge amplifier and recorder (PowerLab, ADInstruments, Colorado Springs, CO, U.S.A.) via in-line transducers (Transpac, Abbott, Abbott Park, IL, U.S.A.) corrected daily for transducer voltage drift and normalized to intra-amniotic pressure. Hemodynamic variables were extracted from the early morning period. Heart rate was determined from the arterial waveform. On days 0, 4, and 8, an arterial blood sample was run on a Radiometer ABL 825 (Radiometer America, Cleveland, OH) for pH, arterial partial pressure of carbon dioxide (PCO_2_), PO_2_, total hemoglobin, oxygen (O_2_) content, oxy-hemoglobin (O_2_-Hb) saturation, glucose, and lactate. Hematocrit was assessed by microcapillary centrifugation, and plasma protein by refractometry, and plasma was collected with heparin and EDTA anticoagulation into a −80°C freezer.

Infusions were initiated or adjusted following assessment of fetal hemodynamics and arterial blood sampling. Experimental fetuses were selected from each twin pair at random, the other was assigned to the Control group. By chance, the Control group included two females and six males, while the experimental group included six females and three males (not different by Fisher’s exact test*, P=*0.1534). Experimental fetuses received Intralipid 20® at an initial dose of 0.5–1 g kg^-1^ d^-1^, increasing daily by 0.5–1 g kg^-1^ d^-1^ to a maximum of 3 g kg^-1^ d^-1^, which is the manufacturer’s recommendations for premature human infants [4]. Infusions were based on fetal weight predicted by age [29]. Actual doses were calculated following necropsy to be initially 0.6 ± 0.2 g kg^-1^ d^-1^, 2.3 ± 0.4 g kg^-1^ d^-1^ by the fourth experimental day, and 2.6 ± 0.4 g kg^-1^ d^-1^ on the final day. Control fetuses received Lactated Ringer’s Solution at an equal volume to the Intralipid infusion rate of their twin. Gestational age on day 0 was 89.0 ± 0.7 dGA (comparable with approximately 25 weeks post conception in humans) and on the final experiment (day 8) was 97.0 ± 0.7 dGA. The eight-day infusion period was selected to meet the duration criteria for many clinical studies of parenteral nutrition outcomes in premature infants [21].

Ewes were humanely killed with an intravenous overdose of a commercial sodium pentobarbital solution. Bolus doses of 10 ml heparin (anti-coagulant) and 10 ml saturated KCl were given via the umbilical vein to arrest the fetal heart in diastole. Fetal weight and sex were recorded. Biopsies were collected from the left midventricular free wall, the right rostral lung lobe, the rostral portion of the left liver lobe, and a B-type placentome (or an A-type if a B-type was not present) for cryopreservation in Tissue-Tek OCT (Optimal Cutting Temperature Media, Fisher Sci, cat: 23–730-571).

### Assessment of plasma lipids

Plasma lipid analysis was performed from heparin-anticoagulated plasma samples in the laboratory as previously described [28]. Intra-assay coefficients of variance for cholesterol, phospholipids, and triglycerides were 2.16%, 5.96%, and 3.06%, respectively. Inter-assay coefficients for cholesterol, phospholipids, and triglycerides were 5.49%, 15.27%, and 2.51%, respectively.

### Fetal circulating hormones

Fetal hormones were measured from EDTA-anticoagulated plasma samples by the Endocrine Technologies Core of the Oregon National Primate Research Center using validated assays as previously described [28]. Values less than the limit of detection were reported as half-way between the limit of detection and zero. Values above the upper threshold of quantification were reported for norepinephrine, entailing a specialized statistical approach. Insulin intra-assay coefficient of variance was 3.70%, inter-assay coefficient of variance was 16.21%; five samples were below the 0.25 ng ml^-1^ limit of detection. Insulin-like growth factor 1 (IGF-1) intra-assay coefficient of variance was 0.72%, and inter-assay coefficient of variance was 15.78%. Insulin-like growth factor 2 (IGF-2) intra-assay coefficient of variance was 3.45%, and inter-assay coefficient of variance was 5.15%. Norepinephrine intra-assay coefficient of variance was 14.24%, and inter-assay coefficient of variance was 13.55%. There were no values below the limit of detection for IGF-1, IGF-2, or norepinephrine.

### Assessment of fetal liver function

A fetal liver panel was measured in frozen heparin-anticoagulated plasma collected on the final study day by the clinical Core Lab at Oregon Health & Science University as previously described [28].

### Oil Red O staining

Tissues were sectioned at 10 μm on a Leica CM3050S cryostat as previously described [23,24]. Tissue sections from experimental animals and positive controls were mounted onto Leica Apex clipped corner microscope slides and kept at −80°C until staining. To stain for Oil Red O, slides were brought to room temperature for 15 minutes and fixed in 10% Neutral Buffered Formalin for 1 minute. Slides were incubated in double-filtered Oil Red O solution (0.5% w/v Oil Red O in isopropanol and diluted 3:2 in distilled water) for 10 minutes and rinsed in tap water. They were then counterstained with hematoxylin (Epic Scientific, cat: HEM-C with 4.17% v/v glacial acetic acid) for 1 minute, incubated in bluing (Epic Scientific, BLU-C) for 1 minute, and mounted with 1:1 diluted Clear- Mount (Electron Microscopy Sciences cat: 1798515) until dry. Slides were dipped in xylenes to soften the Clear-Mount surface, and then coverslipped on a Leica CV5030 automatic coverslipper. Images were captured using a Leica AT2 slide scanner (Leica Biosystems Imaging, Inc.) controlled by Leica ScanScope Scanner Console (Version 102.0.7.5), using an Olympus Plan Apo 20 x / 0.75 NA air objective with a 503.1 nm/pixel XY resolution. Images were saved in Tiled TIFF format (SVS) for image analysis. Images were coded to obscure treatment groups. Low power scan was used to confirm relative uniformity of staining. Images were scored by a pathologist (AC) according to criteria developed and validated to diagnose milk aspiration in pulmonary macrophages from pediatric bronchoalveolar lavage samples [20]. Scoring was performed using Leica ImageScope (Version 12.4.6.5003) in 100 parenchymal cells at the ‘20 ×’ setting in a random but representative field, then in another 100 cells in 1–3 more non-contiguous random but representative fields (avoiding tissue edges and folded tissues). Scores were summed within each field and averaged between fields. The number of scored tissues is reduced by tissues lost after initial analysis.

### Statistics

Significance was defined at *α* = 0.05, except where noted in the text α was corrected for multiple comparisons. Continuous variables are summarized as mean ± SD.

Fisher’s exact test was used to assess dichotomous distribution of sexes. Oil Red O scores were analyzed by Mann–Whitney after visual assessment to determine similarity of treatment response by sex. These analyses were carried out in GraphPad Prism (v.10.4.1).

Shapiro–Wilk’s test was used to assess normality of data (*P*>0.05). Levene’s test was used to assess homogeneity of variances. Outliers were defined as 1.5 box lengths or more from the edge of the box in a boxplot. Fetal weights and liver panel parameters were analyzed by two-way ANOVA (by sex and treatment). Hemodynamic and arterial chemistry parameters were analyzed by mixed three-way ANOVA (by day, sex, and treatment). The Greenhouse–Geisser correction for sphericity was used when there were more than two repeated measures. If the interaction term was significant, main effects were not considered. Multiple comparisons with the Šidák correction were performed if indicated. These statistical analyses were carried out in SPSS (v.30.0.0.0).

Norepinephrine at time point 8 was compared with time point 0 using a two-sided Exact Wilcoxon–Pratt Signed-Rank Test, while Control was compared with Intralipid within age using Exact Wilcoxon–Mann–Whitney Test (ties with mid-ranks method). These analyses were performed with the ‘coin’ package in R (v 4.4.0), testing the null hypothesis that the distribution of differences between paired observations is symmetric around 0. Family-wise significance was determined at *α* = 0.0125.

## Results

### Circulating fetal plasma lipids

**Figure 1:**
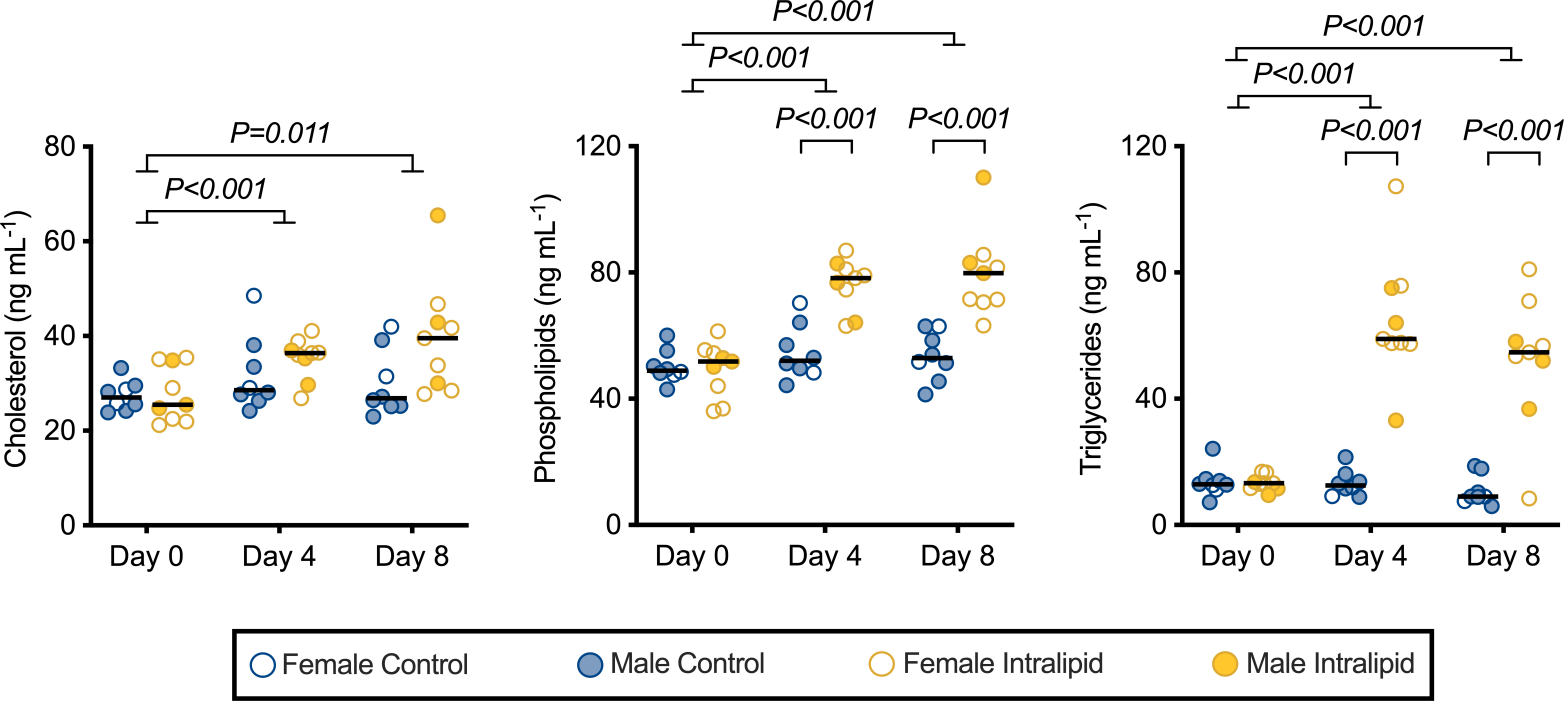
Plasma lipid response to intravenous Intralipid. Fetal plasma cholesterol, phospholipid, and triglyceride levels in fetuses receiving intravenous Intralipid or Lactated Ringer’s Solution. Number for Control female = 2, male = 6; Intralipid female = 6, male = 3. Individual data points shown with bar representing mean (sexes combined). Mixed measures three-way ANOVA (within factors by day, and between factors by treatment and sex). If indicated, multiple comparisons made with the Šidák correction (see Tables 1 and 2).

Circulating fetal plasma lipid levels and statistical analysis are shown in Figure 1 and Tables 1 and 2. Prior to experimental infusions, plasma lipids were similar between the groups (cholesterol 27.62 ± 4.70 mg dl^-1^; phospholipids 49.73 ± 6.97 mg dl^-1^; triglycerides 13.49 ± 3.58 mg dl^-1^). At day 4, Intralipid infusion increased plasma cholesterol and phospholipids by 50% (cholesterol: vs. day 0 *P*<0.001; phospholipids: vs. day 0 *P=*0.001 and vs. Control *P*<0.001) and triglycerides were increased four-fold (vs. day 0 and vs. Control *P*<0.001). Plasma lipid concentrations remained elevated but did not increase further at day 8 (vs. day 0 *P=*0.011 for cholesterol; vs. day 0 and vs. Control *P*<0.001 for triglycerides and phospholipids).

**Table 1:** Mean and standard deviation for fetal plasma lipids

	Day 0		Day 4		Day 8	
	Control	Intralipid	Control	Intralipid	Control	Intralipid
Cholesterol (mg dl^-1^)	27.40 ± 3.16	27.81 ± 5.94	31.94 ± 8.02	35.29 ± 4.40	29.97 ± 7.01	39.61 ± 11.86
Phospholipids (mg dl^-1^)	50.28 ± 5.16	49.25 ± 8.57	54.77 ± 8.70	76.32 ± 8.02*	53.55 ± 7.78	79.68 ± 13.53*
Triglycerides (mg dl^-1^)	13.07 ± 1.18	13.34 ± 2.08	12.62 ± 2.35	60.35 ± 14.29*	10.60 ± 4.09	54.70 ± 10.06*

Number for Control = 8 (female = 2, male = 6), Intralipid = 9 (female = 6, male = 3). Asterisks indicate significant within-day difference from the Control group by multiple comparisons (*P*<0.025) following mixed measures three-way ANOVA (see Table 2).

**Table 2:** *P*-values (and *F* statistics) from analysis of hemodynamic and arterial blood chemistry parameters, and circulating hormones

	Three-way interaction	Two-way interactions			Main effects			Subgroup data characteristics		
	**Treatment × Sex × Day**	**Test only if three-way interaction is NS.**			**Test only if two- and three-way interactions are NS.**			**Normal distribution** ^**a**^	**Homogeneity of variances**	**Outliers**

		**Treatment × Sex**	**Treatment × Day**	**Sex × Day**	**Treatment**	**Sex**	**Day**			
Plasma cholesterol	0.155 (2.126)	0.147 (2.373)	0.132 (2.357)	0.305 (1.217)	0.251 (1.443)	0.596 (0.295)	0.003^i^ (9.148)	3/4^b^	3/3	2^d^
Plasma phospholipids	0.156 (2.085)	0.219 (1.666)	< 0.001^e^ (12.227)	0.198 (1.771)	-	-	-	4/4	2/3^c^	1^d^
Plasma triglycerides	0.792 (0.213)	0.381 (0.823)	< 0.001^f^ (22.691)	0.863 (0.131)	-	-	-	3/4^b^	2/3^c^	4^d^
Arterial pressure	0.330 (1.030)	0.202 (1.824)	0.328 (1.042)	0.633 (0.240)	0.597 (0.294)	0.613 (0.269)	0.872 (0.027)	4/4	0/2^c^	2^d^
Venous pressure	0.361 (0.903)	0.747 (0.109)	0.809 (0.061)	0.491 (0.505)	0.778 (0.083)	0.611 (0.272)	0.001^j^ (17.614)	3/4^b^	2/2	0
Heart rate	0.487 (0.511)	0.125 (2.693)	0.184 (1.973)	0.320 (1.071)	0.390 (0.792)	0.969 (0.002)	0.002^k^ (15.547)	4/4	1/2^c^	2^c^
pH	0.861 (0.032)	0.265 (1.380)	0.467 (0.567)	0.683 (0.176)	0.186 (1.987)	0.018 l (7.701)	0.339 (0.999)	3/4^b^	2/2	2^d^
Hematocrit	0.199 (1.834)	0.109 (2.960)	0.337 (0.996)	0.447 (0.615)	0.451 (0.604)	0.870 (0.028)	0.001^m^ (17.093)	4/4	2/2	0
Total hemoglobin	0.123 (2.788)	0.134 (2.619)	0.258 (1.422)	0.383 (0.825)	0.294 (1.216)	0.776 (0.085)	0.002^n^ (15.259)	4/4	2/2	1^d^
PCO_2_	0.860 (0.033)	0.580 (0.325)	0.994 (0.000)	0.907 (0.014)	0.372 (0.867)	0.345 (0.972)	0.806 (0.064)	4/4	2/2	2^d^
PO_2_	0.324 (1.068)	0.226 (1.646)	0.408 (0.741)	0.449 (0.615)	0.522 (0.438)	0.354 (0.937)	0.070 (4.023)	4/4	2/2	4^d^
O_2_-Hb saturation	0.165 (2.210)	0.654 (0.212)	0.340 (0.995)	0.657 (0.209)	0.529 (0.422)	0.367 (0.885)	0.002^o^ (15.723)	4/4	2/2	3^d^
O_2_ content	0.224 (1.658)	0.181 (2.041)	0.301 (1.177)	0.307 (1.147)	0.323 (1.069)	0.582 (0.322)	0.008^p^ (10.666)	4/4^b^	2/2	2^d^
Plasma protein	0.146 (2.387)	0.073 (3.816)	0.965 (0.002)	0.829 (0.049)	0.598 (0.292)	0.839 (0.043)	< 0.001^q^ (25.772)	3/4^b^	2/2	4^d^
Glucose	0.432 (0.691)	0.613 (0.272)	0.054 (4.672)	0.351 (0.948)	0.384 (0.822)	0.068 (4.083)	0.045^r^ (5.091)	3/4^b^	1/2^c^	1^d^
Lactate	0.309 (1.138)	0.654 (0.212)	0.025^g^ (6.712)	0.882 (0.023)	-	-	-	3/4^b^	2/2	1^d^
Insulin	0.115 (2.858)	0.652 (0.213)	0.022^h^ (6.771)	0.195 (1.870)	-	-	-	4/4	2/2	0
IGF-1	0.588 (0.308)	0.409 (0.729)	0.273 (1.309)	0.638 (0.232)	0.529 (0.418)	0.457 (0.588)	0.007^s^ (595.202)	4/4	2/2	0
IGF-2	0.931 (0.008)	0.879 (0.024)	0.778 (0.083)	0.748 (0.107)	0.616 (0.264)	0.566 (0.347)	0.823 (0.052)	4/4	2/2	0

Number for Control=8 (female=2 except pressures =1 (values missing due to catheter failure), male = 6), Intralipid = 9 (female = 6, male = 3). Analysis by mixed measures three-way ANOVA (factors: treatment, sex, study day). The Greenhouse–Geisser correction for sphericity was used for comparisons with three levels of repeated measures (plasma lipids). For measures with only two repeated values (by study day), there was no correction for sphericity. Not significant (NS).

(a) Normality could not be assessed for Control females as n=2 (Control female pressures n =1; value missing due to catheter failure).

(b) Indicated proportion of subgroups was normally distributed. Although ANOVAs are fairly robust to deviations from normality, interpret results with caution.

(c) Only indicated proportion of subgroups had homogeneity of variances. Although ANOVAs are fairly robust to heterogeneity of variance, interpret results with caution.

(d) Number of outliers found and determined to be biologically relevant and included in analysis (out of N=34).

Simple main effects were assessed following significant Treatment x Day interaction and were assessed using the Šidák correction and family-wise significance of P<0.025:

(e) Control vs Intralipid day 4 = 0.001, day 8 <0.001; Intralipid day 0 vs. day 4 <0.001, day 0 vs. day 8 <0.001.

(f) Control vs. Intralipid day 4 <0.001, day 8 <0.001; Intralipid day 0 vs. day 4 <0.001, day 0 vs. day 8 <0.001.

(g) A treatment effect was found on day 8 (P=0.009, F = 10.173). Cohen’s d (effect size) was 1.30 (‘large’).

(h) No significant treatment effect was found (day 8: P=0.058, F = 4.312, Cohen’s d = 0.89).

Cohen’s d (effect size):

(i) day 0 vs. day 4 <0.001, Cohen’s d=0.96 (‘large’); day 0 vs. day 8 = 0.011, Cohen’s d = 0.83 (‘large’)

(j) 1.01 (“large”); (k) 1.02 (“large”); (l) 0.87 (“large”); (m) 1.00 (“large”); (n) 1.04 (“large”); (o) 1.16 (“large”); (p) 0.90 (“large”); (q) 1.26 (“large”); (r) 0.10 (less than “small”); (s) 1.30 (“large”)

### Fetal hemodynamics and arterial blood chemistry

**Figure 2:**
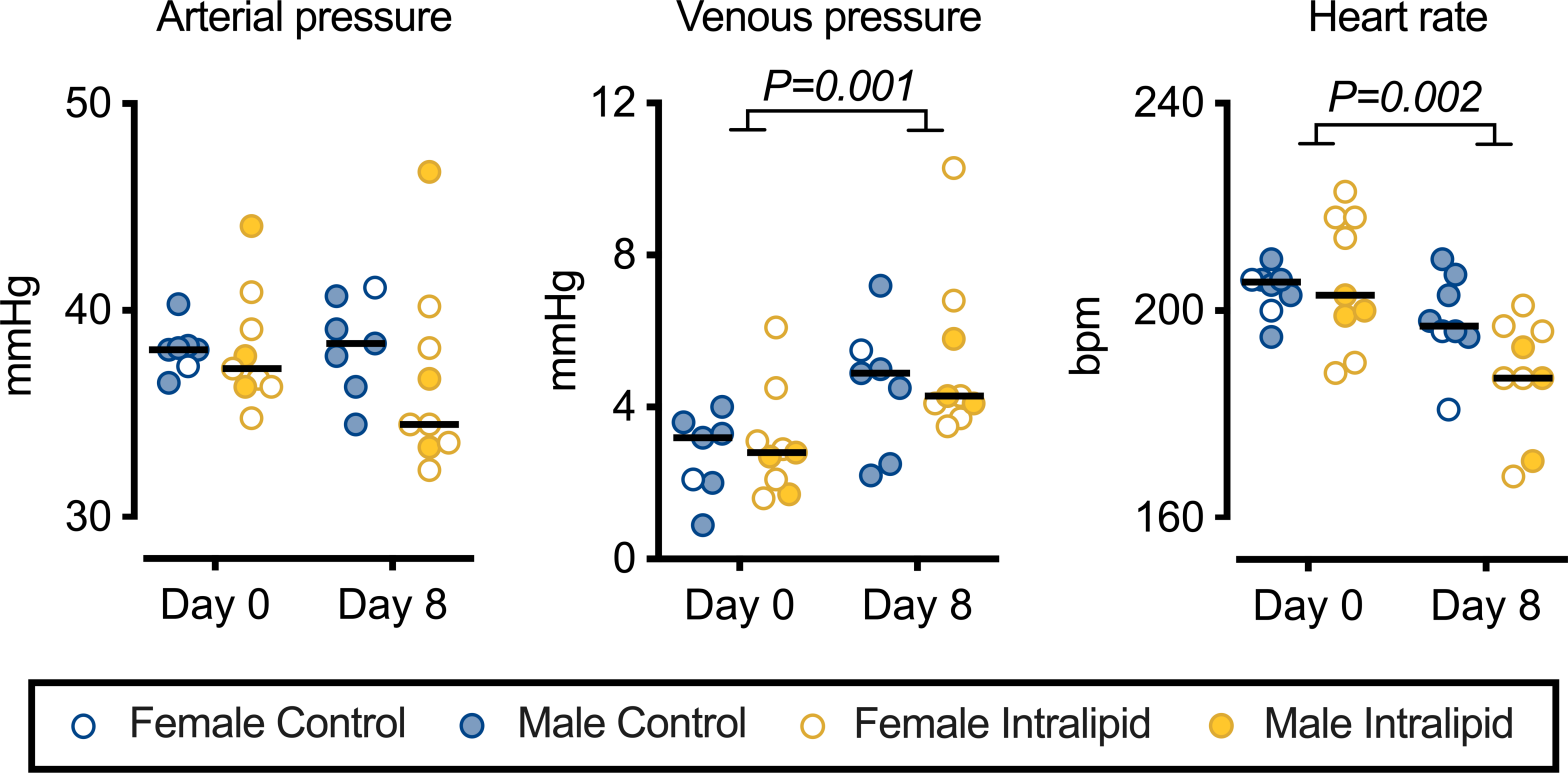
Fetal hemodynamic response to intravenous Intralipid. Central arterial and venous pressure and heart rate in fetuses receiving intravenous Intralipid or Lactated Ringer’s Solution. Number for Control female = 2 (except for arterial and venous pressures = 1 due to a catheter failure), male = 6; Intralipid female = 6, male = 3. Individual data points shown with bar representing mean (sexes combined). Mixed measures three-way ANOVA (within factors by day, and between factors by treatment and sex). If indicated, multiple comparisons made with the Šidák correction (see Tables 2 and 3).

Fetal hemodynamic parameters and statistical analysis are shown in Figure 2 and Tables 2 and 3, and there were no significant differences between groups in arterial blood pressure. Between days 0 and 8, independent of treatment or sex, venous blood pressure increased by 63% (*P=*0.001), and heart rate declined by 6% (*P=*0.002).

**Table 3:** Mean and standard deviation for fetal hemodynamics, blood gases, and hormone concentrations

	Day 0		Day 8	
	Control	Intralipid	Control	Intralipid
Arterial pressure (mmHg)	38.1 ± 1.2	38.1 ± 2.9	38.3 ± 2.3	36.7 ± 4.8
Venous pressure (mmHg)	2.7 ± 1.1	3.1 ± 1.4	4.5 ± 1.7	5.2 ± 2.2
Heart rate (min^-1^)	204 ± 5	206 ± 13	198 ± 9	187 ± 11
pH	7.372 ± 0.020	7.366 ± 0.021	7.374 ± 0.015	7.376 ± 0.013
Hematocrit (%)	30.5 ± 3.5	30.1 ± 3.6	40.0 ± 9.6	37.0 ± 9.5
Total hemoglobin (g dl^-1^)	9.7 ± 0.8	9.5 ± 1.0	13.7 ± 4.1	12.4 ± 3.7
PCO2 (mmHg)	48.2 ± 2.4	49.0 ± 3.6	48.2 ± 1.8	48.6 ± 2.5
PO2 (mmHg)	27.2 ± 2.4	27.6 ± 2.1	30.5 ± 7.0	29.4 ± 5.8
O2-Hb saturation (%)	71.7 ± 4.0	72.1 ± 3.1	64.5 ± 4.5	66.3 ± 6.3
O2 content (ml dl^-1^)	9.4 ± 1.1	9.2 ± 0.6	11.7 ± 3.0	10.8 ± 2.2
Plasma protein (g dl^-1^)	2.6 ± 0.1	2.5 ± 0.2	3.4 ± 0.7	3.4 ± 0.8
Glucose (mmol l^-1^)	1.3 ± 0.3	1.3 ± 0.2	1.2 ± 0.3	1.3 ± 0.2
Lactate (mmol l^-1^)	1.3 ± 0.2	1.1 ± 0.5	1.3 ± 0.2	1.0 ± 0.2*
Insulin (ng m l^-1^)	0.39 ± 0.16	0.40 ± 0.13	0.45 ± 0.16	0.30 ± 0.15
IGF-1 (ng m l^-1^)	31.5 ± 4.9	31.7 ± 5.7	44.1 ± 11.0	36.7 ± 12.0
IGF-2 (ng m l^-1^)	2,263 ± 169	2,333 ± 266	2,278 ± 341	2,278 ± 248
Norepinephrine (pg m l^-1^)	162 ± 55	203 ± 57	174 ± 141	138 ± 107

Number for Control = 8 (female = 2 except pressures = 1; values missing due to catheter failure, male = 6), Intralipid = 9 (female = 6, male = 3). Asterisks indicate significant within-day difference from the Control group by multiple comparisons (*P*<0.025) following mixed measures three-way ANOVA (see Table 2 ).

**Figure 3:**
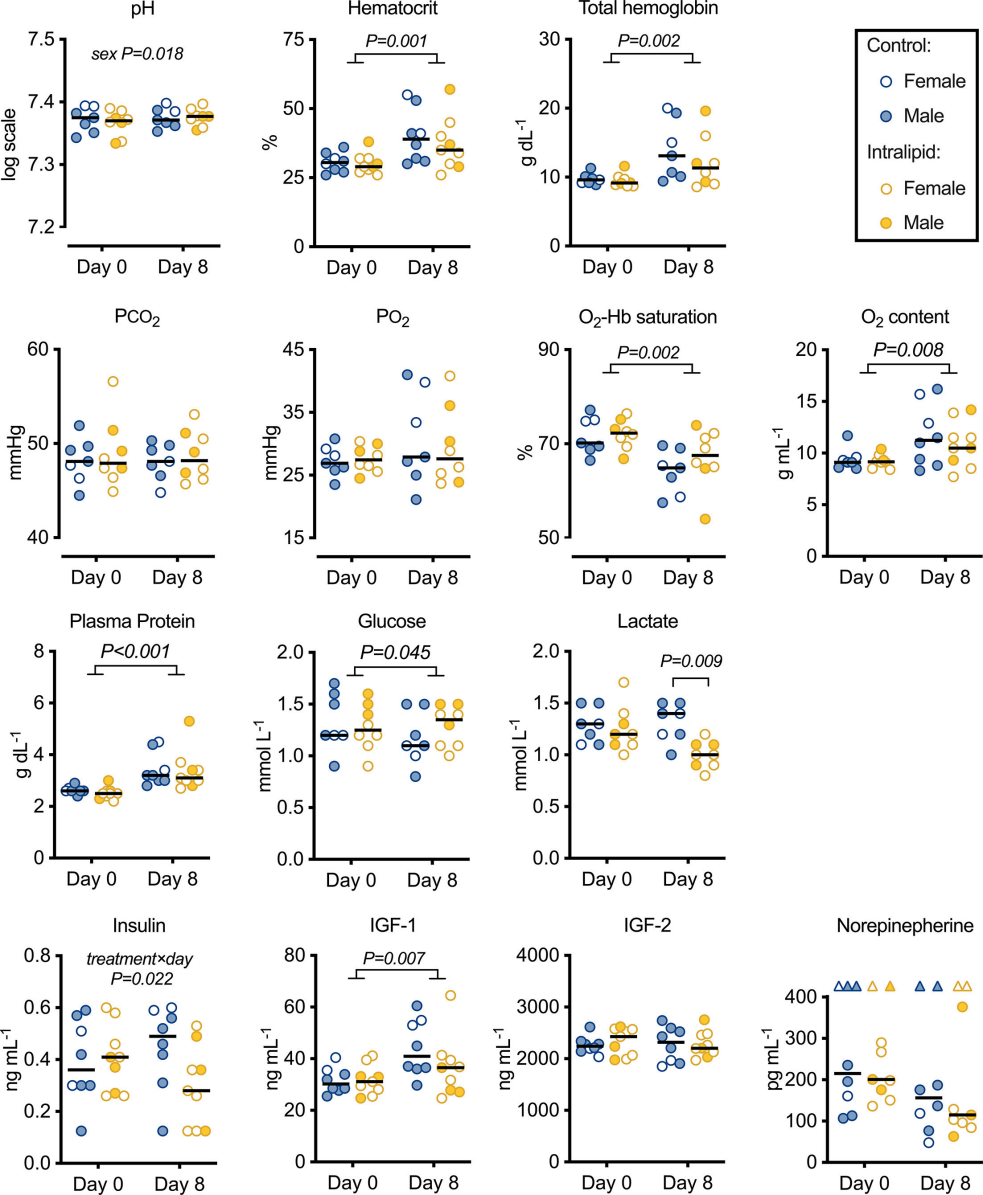
Arterial blood gases and chemistry response to intravenous Intralipid. Fetal arterial pH, plasma protein, hematocrit, total hemoglobin, partial pressure of CO_2_ (PCO_2_), partial pressure of O_2_ (PO_2_), oxy-hemoglobin (O_2_-Hb) saturation, O_2_ content, plasma protein, glucose, lactate, plasma insulin, plasma IGF-1, plasma IGF-2, and plasma norepinepherine in fetuses receiving intravenous Intralipid or Lactated Ringer’s Solution. Number for Control female = 2, male = 6 (except parameters measured on the Radiometer = 5 due to equipment failure); Intralipid female = 6 (except parameters measured on the Radiometer = 5), male = 3. Individual data points shown with bar representing mean (sexes combined). Triangular icons represent values above the upper threshold of quantification. Mixed measures three-way ANOVA (within factors by day, and between factors by treatment and sex). If indicated, multiple comparisons made with the Šidák correction (see Tables 2 and 3). Norepinephrine levels at day 8 were compared with day 0 using a two sided Exact Wilcoxon–Pratt Signed-Rank test with the ‘coin’ package in R (v 4.4.0).

Fetal arterial blood chemistry parameters and statistical analysis are shown in Figure 3 and Tables 2 and 3. Arterial blood pH was 0.0146 units less in male than female fetuses regardless of day or treatment (*P=*0.018). Between days 0 and 8, independent of treatment or sex, there were increases in hematocrit (27%; *P=*0.001), total hemoglobin concentration (36%; *P=*0.002), oxygen content (21%; *P=*0.008), and plasma protein (32%; *P*<0.001). Over the same period, oxyhemoglobin saturation decreased 9% (*P=*0.002) and glucose decreased 2% (*P=*0.045). There was a significant interaction among treatment and day for lactate (*P=*0.025); on day 8, lactate levels were 21% lower in Intralipid-treated fetuses than in Control (*P=*0.009). PCO_2_ and PO_2_ were unchanged by experimental day, treatment, or sex.

### Circulating fetal hormones

Hormones circulating in fetal plasma and statistical analysis are shown in Figure 3 and Tables 2 and 3. There was a significant interaction among treatment and day for insulin (*P=*0.022); no significant differences between subgroups were found. Independent of treatment and sex, IGF-1 levels increased 27% between days 0 and 8 (*P=*0.007). IGF-2 was unchanged by experimental day, treatment, or sex. Norepinephrine was not found to be different between days 0 and 8 (Control *P=*0.2812; Intralipid *P=*0.4258) and not different between treatment groups (day 0 *P=*0.8865; day 8 *P=*0.7631)

### Fetal growth

**Figure 4:**
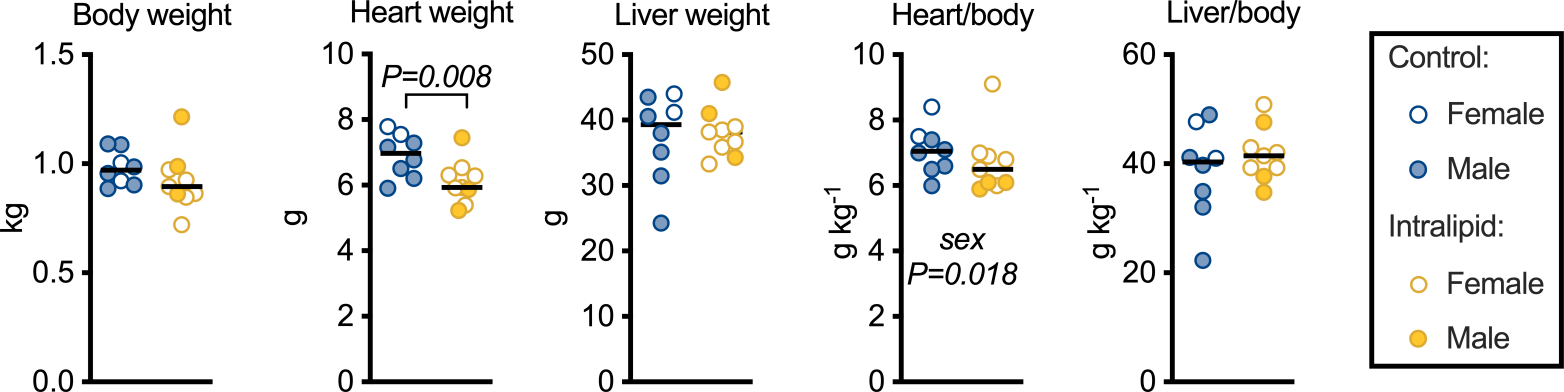
Body and organ weight response to Intralipid infusion. Fetal body, heart, and liver weights were measured following eight days of intravenous Intralipid or Lactated Ringer’s Solution. Number for Control female = 2, male = 6; Intralipid female = 6, male = 3. Individual data points shown with bar representing mean (sexes combined). Analyzed by two-way ANOVA (between factors by treatment and sex; see Tables 4 and 5).

Fetal body and organ weights and statistical analysis are shown in Figure 4 and Tables 4 and 5. Neither eight days of Intralipid infusion nor fetal sex affected terminal body weight, liver weight, or the weight ratio of liver to body. Heart weight was 12% lower in the Intralipid group (*P=*0.008), and the weight ratio of heart to body was 10% lower in males than females, independent of treatment (*P=*0.018).

**Table 4:** Mean and standard deviation for fetal measures obtained on the final study day

	Day 8	
	Control	Intralipid
Body weight (kg)	0.98 ± 0.08	0.92 ± 0.14
Heart weight (g)	6.9 ± 0.7	6.1 ± 0.7*
Liver weight (g)	37.3 ± 6.7	38.1 ± 3.8
Heart/body (g kg^-1^)	7.1 ± 0.8	6.7 ± 1.0
Liver/body (g kg^-1^)	39 ± 9	42 ± 5
Albumin (g dl^-1^)	1.9 ± 0.2	2.0 ± 0.2
Alkaline phosphatase (U l^-1^)	149 ± 57	147 ± 41
Aspartate aminotransferase	9.1 ± 1.8	12.1 ± 4.3
Bilirubin, unconjugated (mg dl^-1^)	0.3 ± 0.1	1.2 ± 0.5*
Bilirubin, conjugated (mg dl^-1^)	0.4 ± 0.1	0.6 ± 0.1*
Globulin (g dl^-1^)	1.4 ± 0.4	1.3 ± 0.5
Liver Oil Red O score	18.9 ± 17.2	371.7 ± 44.2*
Lung Oil Red O score	35.0 ± 17.0	32.1 ± 12.8
Heart Oil Red O score	1.8 ± 2.8	97.6 ± 60.1*
Placenta Oil Red O score	91.5 ± 47.3	117.3 ± 39.7

Number for Control = 8 (female = 2, male = 6), Intralipid = 9 (female = 6, male = 3). Asterisks indicate significant difference from the Control group by multiple comparisons (*P*<0.025) following two-way ANOVA (see Table 5).

**Table 5:** P-values (and F statistics) from analysis of fetal weights and liver function panel

	2-Way interaction	Main effects		Subgroup data characteristics		
		Test only if 2-way interaction is NS.		Normal distribution *a*	Homogeneity of variances	Outliers
	Treatment × Sex	Treatment	Sex			
Body weight	0.278 (1.282)	0.627 (0.248)	0.153 (2.307)	3/3	Yes	1 *d*
Heart weight	0.105 (3.030)	0.008 *f* (9.818)	0.193 (1.886)	3/3	Yes	0
Liver weight	0.076 (3.729)	0.513 (0.452)	0.886 (0.021)	3/3	Yes	0
Heart/body	0.841 (0.042)	0.066 (4.028)	0.018 *g* (7.308)	2/3 *b*	Yes	1 *d*
Liver/body	0.500 (0.482	0.809 (0.061)	0.183 (1.979	3/3	Yes	1 *d*
Albumin	0.036 *e* (5.442)	-	-	3/3	Yes	1 *d*
Alkaline phosphatase	0.487 (0.512)	0.919 (0.011)	0.791 (0.073)	3/3	Yes	1 *d*
Aspartate aminotransferase	0.147 (2.377)	0.171 (2.097)	0.819 (0.055)	3/3	No *c*	1 *d*
Bilirubin, unconjugated	0.908 (0.014)	< 0.001 *h* (18.833)	0.518 (0.442)	3/3 *b*	No *c*	0
Bilirubin, conjugated	0.854 (0.035)	< 0.001 *i* (33.674)	0.213 (1.717)	3/3 *b*	Yes	2 *d*
Globulin	0.133 (2.573)	0.545 (0.387)	0.773 (0.087)	3/3	No *c*	1 *d*

(a) Normality could not be assessed for Control females as n=2 (for Control female pressures n = 1; values missing due to catheter failure).

(b) Only indicated number of subgroups was normally distributed. Although ANOVAs are fairly robust to deviations from normality, interpret results with caution.

(c) Although ANOVAs are fairly robust to heterogeneity of variance, interpret results with caution.

(d) Outliers determined to be biologically relevant and included in the analysis.

(e) Simple main effects were assessed using the Šidák correction and family-wise significance of P<0.025. No significant differences were found.

Cohen’s d (effect size):

(f) 1.05 (‘large’)

(g) 0.87 (‘large’)

(h) 1.58 (‘large’)

(i) 1.70 (‘large’)

### Fetal liver function

**Figure 5:**
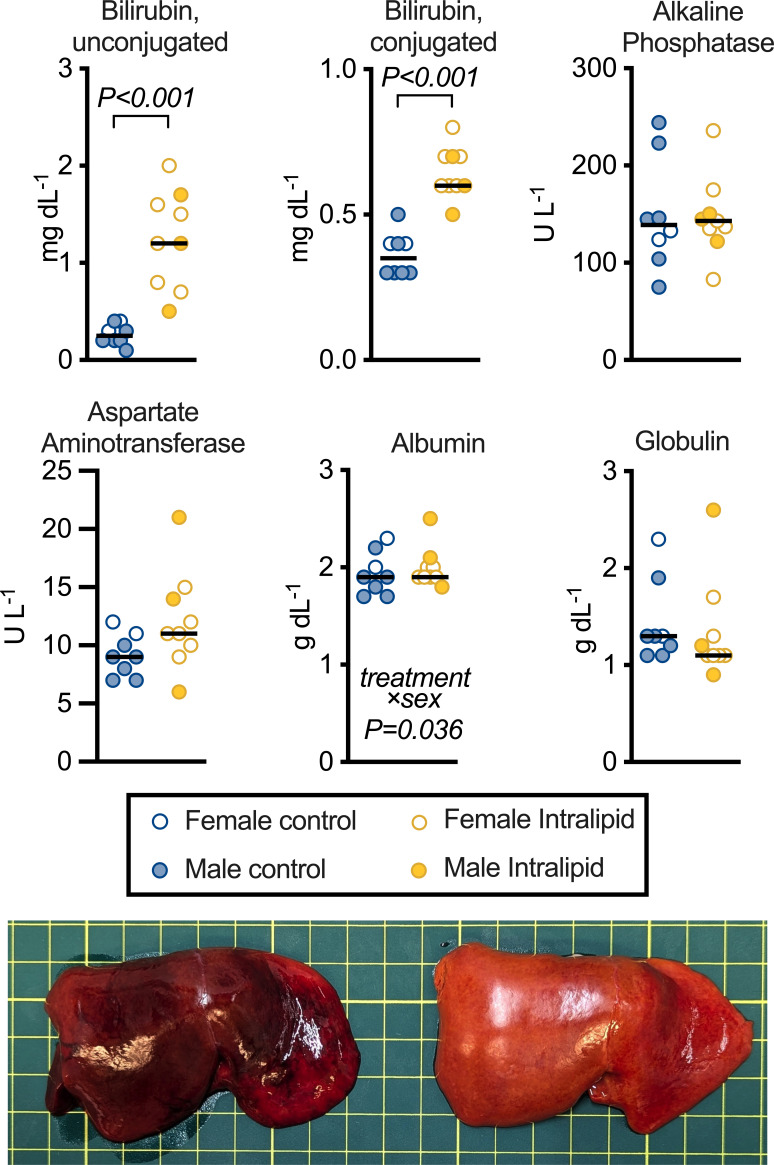
Fetal liver function panel results following Intralipid infusion *Top:* A panel of Liver health markers were measured in fetal plasma on the terminal day. Liver damage may be indicated by elevated bilirubin (although in the fetus the placenta is responsible for elimination of bilirubin), alkaline phosphatase, and aspartate aminotransferase, and by reduced albumin and globulin. Number for Control female = 2, male = 6; Intralipid female = 6, male = 3. Individual data points shown with bar representing mean (sexes combined). Two-way ANOVA (between factors by treatment and sex; see Tables 4 and 5). *Bottom:* Representative livers from a Control (left) and an Intralipid-infused (right) fetus. Each square is 1 cm^2^.

Liver health at the end of the infusion period was assessed by a clinical panel shown in Figure 5 and Table 5 , with statistical analysis in Table 4 . A significant interaction between treatment and sex was found for albumin (*P=*0.036); no significant differences among subgroups were found. Levels of unconjugated bilirubin were more than 3-fold higher in Intralipid-infused fetuses (*P*<0.001). Levels of conjugated bilirubin, which has been processed by the liver, were 78% higher than in Controls (*P*<0.001). Alkaline phosphatase, aspartate aminotransferase, and globulin levels were not shown to be affected by fetal Intralipid infusion or sex.

### Oil Red O staining

**Figure 6:**
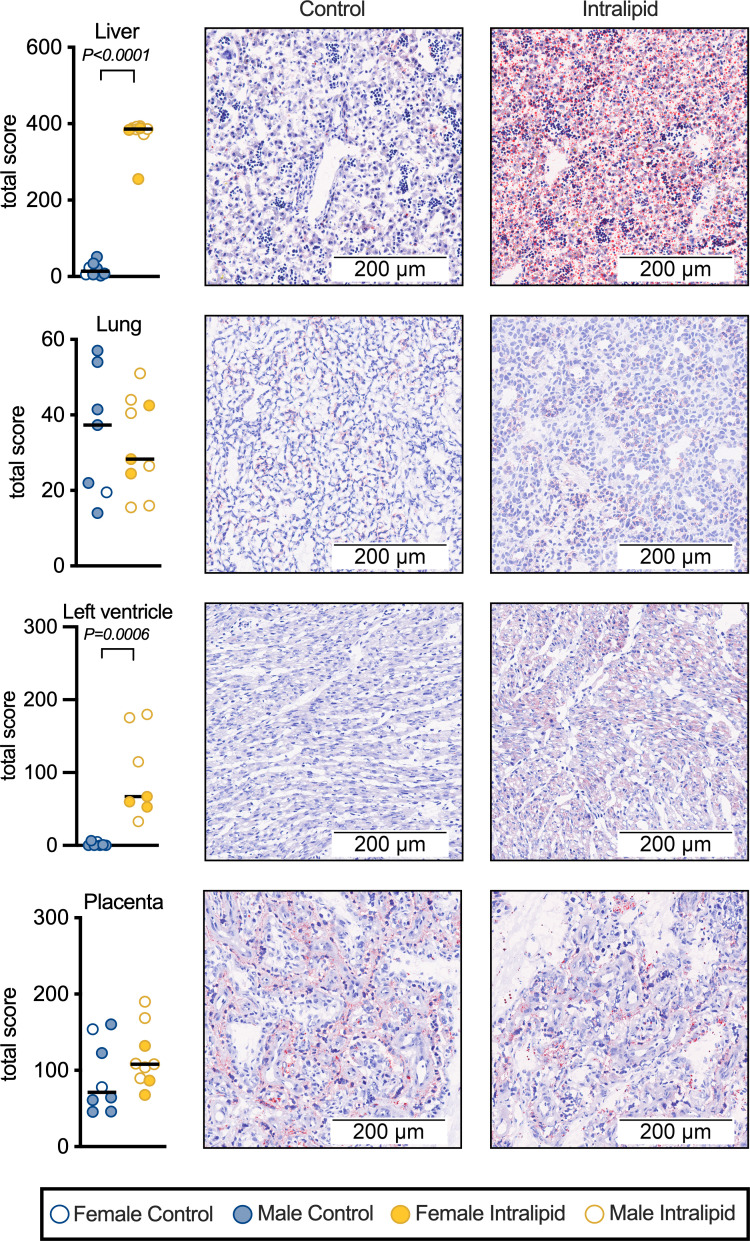
Oil Red O staining response to Intralipid infusion Neutral lipids and lipid droplets accumulated within parenchymal cells were stained with Oil Red O in fetal liver, lung, heart, and placenta. Raw and median values are shown. Number for Control female = 2, male = 6; Intralipid female = 6, male = 3. The treatment effect was visually assessed by sex prior to analysis by Mann–Whitney test.

Neutral lipid and lipid droplet content were assessed by Oil Red O staining. Data and statistical analyses are shown in Figure 6 and Tables 4 and 5. Staining in parenchymal cells of fetal liver was elevated 20-fold by Intralipid administration (*P*<0.0001); qualitatively, the largest droplets may have been in the extracellular space. Lipid staining in the left ventricle was 55-fold higher in Intralipid fetuses compared with Controls (*P=*0.0006). Oil Red O staining in the fetal lung and placenta was unaffected by treatment. In the placenta, Oil Red O staining was found in all cases in both fetal and maternal tissues; qualitatively, the greatest variability was in the trophoblast rather than villous stromal cells or maternal cells.

## Discussion

In the present study, Intralipid was administered to mid-gestation fetal sheep to isolate the physiological response to high plasma lipid concentrations in the absence of other pathological conditions. Our findings suggest that fetuses can tolerate lipid emulsion at an infusion rate of 2.6 g kg^-1^ d^-1^ during this stage of development. We did not detect any changes in fetal hemodynamics or blood gases as a result of the Intralipid treatment. Fetal body weight was similar between treatment groups, but heart weight was lower in mid-gestation fetuses receiving Intralipid. Compared with Controls, Intralipid fetuses also had lower blood lactate levels after eight days of treatment. Liver weight was unaffected by lipid emulsion, but increased bilirubin levels and lipid droplet accumulation may suggest possible impairments in liver function resulting from high circulating lipids. Further, lipid accumulation in the heart of Intralipid-treated fetuses may precede pathological metabolic conditions associated with ectopic lipid storage.

### Plasma lipid regulation during mid-gestation Intralipid infusion

Fetuses are thought to lack the cellular machinery for lipid clearance and utilization, especially during the early stages of development. To evaluate the physiological consequences of high plasma lipids, we continuously infused Intralipid in mid-gestation fetal sheep at a dose comparable with that administered to infants receiving parenteral nutrition. After four days of lipid infusion, fetal plasma triglycerides increased four-fold while cholesterol and phospholipid levels increased by about 50%. Plasma lipid concentrations were consistent from day 4 to 8 despite a gradual increase in Intralipid infusion rate to match fetal growth. The lipid levels achieved on days 4 and 8 of Intralipid treatment are consistent with those of fetuses exposed to maternal dyslipidemia [15] and to very preterm infants receiving intravenous lipid emulsions [10]. However, fetal plasma triglyceride and cholesterol levels remained well below the concentrations associated with hypertriglyceridemia and hypercholesteremia during parenteral nutrition and are not indicative of lipid intolerance [10,30–32].

Despite baseline lipid levels being higher at 87 dGA compared with those previously measured in fetal sheep at 125 dGA [23,24], after 4 and 8 days of Intralipid infusion, plasma lipid concentrations reached values similar to those measured in late-gestation. Thus, the relative fold changes between days 0 and 8 of Intralipid infusion were less in mid-gestation fetuses compared with late-gestation fetal sheep receiving the same Intralipid treatment [23,24]. Other studies report higher fetal concentrations of cholesterol [33–36] and triglycerides [37] earlier in gestation. Changes across gestation may result from immature hepatic function, lower lipase activity, smaller adipose tissue stores, and the absence of intestinal lipid absorption in early- and mid-gestation fetuses [10,31,32,38]. Further, similarities in the lipid levels achieved from Intralipid infusion in mid- and late-gestation suggest that the degree of prematurity does not drive the hypertriglyceridemia reported in extremely low birth weight infants [30]. These results indicate that the capacity for lipid handling and clearance is comparable between mid- and late-gestation fetuses.

### Physiological changes not related to Intralipid

Although Intralipid emulsion significantly altered the lipid composition of the developmental milieu, fetal hemodynamic pressures and blood chemistry parameters were unaffected by the increase in circulating lipids. Instead, we noted several differences between study days 0 and 8 that were independent of treatment or fetal sex. The decline in fetal heart rate is typical of development [39,40], as is the decline in O_2_-Hb saturation, which is likely due to the switch from fetal to adult hemoglobin that begins at 45 dGA [41]. In contrast, the increases in venous pressure, hematocrit, and total Hb are not reflective of normal developmental biology. We postulate that these are a consequence of fetal catheterization at this gestational age, as this occurred in both Intralipid and Control fetuses, and we have observed it in other fetal groups catheterized at the same gestational age. Notably, in fetuses excluded due to hydrops fetalis or fetal demise, we noted a rapid rise in hematocrit and venous pressure. This finding is of unknown etiology as we confirmed that the venous catheter tip was almost always, including in those cases, distal to the thoracic duct. Regardless of the cause, these observations support the elevated venous pressure on study day 8 being related to fetal surgery or catheterization. Further, the increase in venous pressure may have stimulated transudation and thus increased hematocrit, with elevated total Hb increasing O_2_ content despite reduced O_2_-Hb saturation. Our previous study in fetuses at a later stage of development did not reveal any sex-related differences in fetal arterial pH [23,24]. Thus, the higher pH in female fetuses found in this study may be a type I statistical error, biological variability, or an effect dependent on gestational age.

### Hormone modulation by mid-gestation Intralipid infusion

The nutrient and hormone profiles observed in the present study indicate that Intralipid treatment during mid-gestation altered the fetal metabolic milieu. We measured a decrease in circulating glucose levels with advancing gestational age, independent of treatment or fetal sex. This trend is consistent with normal development [1,42,43] and aligns with the lower glucose concentrations reported in fetal sheep at 125 dGA [23,24]. Fetal IGF-1 levels were elevated across treatment groups on the final experimental day, while IGF-2 concentrations remained unchanged by Intralipid exposure or treatment day. This pattern is consistent with known developmental trends, as IGF-1 typically increases during mid-gestation, whereas IGF-2 remains relatively stable throughout fetal development [23,24,44–47]. The reduction in fetal lactate levels following Intralipid infusion may reflect a decrease in glucose metabolism [42], likely driven by a shift toward fatty acid oxidation in response to increased lipid availability. This is supported by the interaction between treatment and day for circulating insulin levels, as the tendency toward reductions in plasma insulin after Intralipid infusion may limit cellular glucose uptake and utilization [48,49]. These findings challenge previous assumptions that fetuses at this stage of development lack the capacity for fatty acid oxidation [2–4]. However, further research is needed to assess the proposed shifts in fetal metabolism.

### Fetal growth during mid-gestation Intralipid infusion

Total body weight was similar between Intralipid and Control fetuses, suggesting that overall fetal growth is not impacted by short-term increased circulating lipid concentrations. However, because we did not assess fetal growth trajectories beyond the eight-day treatment period, it remains unclear whether these effects would persist later in gestation. The reduced heart weight in response to Intralipid infusion may reflect an increased vulnerability of the heart to changes in lipid availability during mid-gestation. Further research is needed to understand the cardiac growth response to elevated lipid exposure at this stage of development.

### Lipid deposition during mid-gestation Intralipid infusion

Tissue concentration of intracellular lipids was assessed by Oil Red O staining in the liver, lung, heart, and placenta. High circulating lipids in mid-gestation led to neutral lipid accumulation in the liver and the heart. We speculate that organ-specific expression of lipid transporters and the cellular components necessary for lipid droplet formation are responsible for the differences in organ lipid accumulation. The fold change in lipid droplet score stimulated by Intralipid infusion was notably different between mid-gestation and late gestation [23,24]. For the heart, the change in accumulated lipid during Intralipid infusion was more comparable: 55-fold in late gestation versus 39-fold in mid-gestation. Lung lipid accumulation was not different between groups in this study, but in late gestation, Intralipid treatment increased the lung Oil Red O score. A key maturational characteristic of the late gestation lung is surfactant production, which involves lipid uptake and synthesis [50] and may contribute to the differences in lipid accumulation seen between the fetuses in this study and later gestation fetuses [23,24,51].

In late gestation, Intralipid infusion caused the liver Oil Red O score to double, but in mid-gestation, the score increased 20-fold. The liver has a central role in triglyceride clearance, and fetal hyperlipidemia is closely linked to hepatic dysfunction [52]. While the blanched appearance of the Intralipid liver may reflect hepatic lipid accumulation, plasma markers of liver function suggest that overall liver health was preserved. In contrast to our previous findings of increased liver weight in late-gestation fetuses treated with Intralipid [23,24], the present study found no difference in liver weight between Control and Intralipid fetuses. Circulating albumin levels were similar between groups and plasma protein increased over the treatment period in both experimental and Control fetuses in mid-gestation. In this study, we likely captured the exponential increase in total protein that occurs at the start of the second half of gestation [53], as the trajectory from 89 to 97 dGA would produce much higher protein levels than those measured at 125 dGA [23,24]. Fetuses receiving Intralipid treatment had elevated levels of conjugated and unconjugated bilirubin, which in the postnatal period may be indicative of liver damage due to lipid accumulation. These mid-gestational fetal results are consistent with our previous findings in near-term fetuses [23,24], though bilirubin levels increased to a lesser extent in mid-gestation. While elevated total and conjugated bilirubin can be linked to cholestasis and steatosis in the liver, this is more likely related to the rate of placental clearance of bilirubin from fetal circulation [52,54]. Exposure time to lipids was likely too short to develop steatohepatitis, and no signs were detected. All livers contained yellow pigment that could be bile pigment, although it was easier to see in livers without much lipid staining. There were no signs of lobular inflammation/activity in livers regardless of Oil Red O content, and there was a lot of (normal) extramedullary hematopoiesis, which makes the lobules very hypercellular.

### Limitations of the study

This study is the first to isolate the physiological response of mid-gestation fetuses to elevated lipid levels without the confounding effects of preterm birth or maternal dyslipidemia. The eight-day infusion period was selected to meet the duration criteria for many clinical studies of parenteral nutrition outcomes in premature infants [21], although this short period is less relevant to maternal dyslipidemia. This study gives insights primarily into the acute fetal response to high lipids during a critical mid-gestation window. A limitation of the model was that Control and Intralipid group assignment could not be balanced for fetal sex; however, the ratio of male to female fetuses was not statistically different between groups. Another limitation of this study was that our infusions included heparin, which stimulates release of lipoprotein lipase and reduces likelihood of hypertriglyceridemia [30]. Finally, we only evaluated the effects of Intralipid 20®, although various forms of lipid emulsion are used for parenteral feeding. Further research is needed to assess the short and long-term effects of alternative lipid emulsions, such as SMOF and other olive- or fish oil-based formulations [55–57].

**Clinical Perspectives**

- Intralipid emulsion may be harmful if administered before developmentally appropriate, but prior studies cannot distinguish the effects of elevated lipid exposure from other pathological consequences associated with maternal dyslipidemia or very preterm birth. Therefore, we evaluated the physiological response to intravenous lipid infusion in mid-gestation fetal lambs at the human equivalent of 26 weeks of gestation.
- While hemodynamic and blood chemistry parameters were maintained, intravenous lipid emulsion altered the fetal metabolic environment, elevating plasma lipid concentrations, lowering lactate levels, and increasing lipid accumulation in the liver and heart.
- At 61–66% gestation, the fetus is relatively tolerant of intravenous lipid emulsion and responds similarly to the near-term fetus; however, ectopic lipid accumulation during early development may increase the risk for metabolic complications later in life.

## Data Availability

Data are available upon reasonable request of the corresponding author.

## Open Access

This article has been published open access under our Subscribe to Open programme, made possible through the support of our subscribing institutions, learn more here: https://portlandpress.com/pages/open_access_options_and_prices#conditional

## Abbreviations

Hb: hemoglobin
IGF-1: Insulin-like growth factor 1
IGF-2: Insulin-like growth factor 2
O_2_-Hb: oxy-hemoglobin
PCO_2_: partial pressure of carbon dioxide
PO_2_: partial pressure of oxygen
dGA: days of gestational age
